# Anticonvulsant Action and Long-Term Effects of Chronic Cannabidiol Treatment in the Rat Pentylenetetrazole-Kindling Model of Epilepsy

**DOI:** 10.3390/biomedicines10081811

**Published:** 2022-07-28

**Authors:** Zsolt Gáll, Krisztina Kelemen, Andrea Tolokán, István Zolcseak, István Sável, Réka Bod, Elek Ferencz, Szende Vancea, Melinda Urkon, Melinda Kolcsár

**Affiliations:** 1 Department of Pharmacology and Clinical Pharmacy, George Emil Palade University of Medicine, Pharmacy, Science, and Technology of Targu Mures, 540142 Târgu Mureș, Romania; tolokan.andrea@gmail.com (A.T.); istvanzolcseak@gmail.com (I.Z.); savelistvan@gmail.com (I.S.); melinda.urkon@umfst.ro (M.U.); melinda.kolcsar@umfst.ro (M.K.); 2 Department of Physiology, George Emil Palade University of Medicine, Pharmacy, Science, and Technology of Targu Mures, 540142 Târgu Mureș, Romania; krisztina.kelemen@umfst.ro (K.K.); bod.reka-barbara@stud16.umftgm.ro (R.B.); 3 Department of Physical Chemistry, George Emil Palade University of Medicine, Pharmacy, Science, and Technology of Targu Mures, 540142 Târgu Mureș, Romania; elek.ferencz@umfst.ro (E.F.); vancsa.szende@gmail.com (S.V.)

**Keywords:** cannabidiol, epilepsy, animal model, cognitive dysfunction

## Abstract

Cannabidiol (CBD) showed anticonvulsant action in several preclinical models and is currently approved by regulatory agencies to treat childhood epilepsy syndromes. However, CBD treatment has limited benefits, and its long-term effects on cognition are not fully understood yet. This study aimed to examine the impact of long-term CBD treatment in the pentylenetetrazole (PTZ)-kindling model of epilepsy. Adult male Wistar rats (*N* = 24) received PTZ (35 mg/kg intraperitoneally) every other day until two consecutive generalized seizures occurred. CBD (60 mg/kg body weight) was administered daily by the oral route until the kindled state was achieved (*n* = 12). To confirm that the formulation and administration techniques were not of concern, liquid chromatography–mass spectrometry was performed to test the brain penetration of the CBD formula. As a result of CBD treatment, a lower mortality rate and significantly prolonged generalized seizure latency (925.3 ± 120.0 vs. 550.1 ± 69.62 s) were observed, while the frequency and duration of generalized seizures were not influenced. The CBD-treated group showed a significant decrease in vertical exploration in the open field test and a significant decrease in the discrimination index in the novel object recognition (NOR) test (−0.01 ± 0.17 vs. 0.57 ± 0.15, *p* = 0.04). The observed behavioral characteristics may be connected to the decreased thickness of the stratum pyramidale or the decreased astrogliosis observed in the hippocampus. In conclusion, CBD treatment did not prevent kindling, nor did it affect seizure frequency or duration. However, it did increase the latency to the first seizure and decreased the prolonged status epilepticus-related mortality in PTZ-kindled rats. The cognitive impairment observed in the NOR test may be related to the high dose used in this study, which may warrant further investigation.

## 1. Introduction

Cannabidiol (CBD), the second most important component of *Cannabis sativa*, has no psychotropic effects and holds low toxicity in both humans and experimental animals. Thus, it has been studied in a wide dose range for potential use in various neurological and psychiatric diseases [1,2,3,4,5]. CBD has a weak affinity for cannabinoid receptors CB1 and CB2, acting as a negative allosteric modulator and inverse agonist, respectively [6]. Besides, it also has an antagonistic effect on GPR55 receptors, partial agonist action on 5-HT1A, and a negative allosteric modulatory effect on opioid receptors (µ and δ) [7]. Ion channels are also important targets for CBD, the most-studied being the transient receptor potential vanilloid type 1 (TRPV1) and ankyrin type 1 (TRPA1), and T-type voltage-gated calcium channels, which are involved in the regulation of Ca^2+^ signaling in the brain [3,8,9]. Therefore, CBD treatment could have numerous beneficial outcomes in neuropsychiatric illnesses; however, at present, the only approved indication for CBD is to treat seizures associated with childhood epilepsy syndromes like Lennox–Gastaut and Dravet syndromes [10,11]. 

CBD has a well-described anticonvulsant effect based on in vitro and in vivo models of epilepsy. In vitro, CBD reduced the amplitude and duration of epileptiform activities induced by low concentrations of magnesium and 4-aminopyridine but did not influence signal propagation [12]. CBD administered in a dose range of 40–360 mg/kg body weight ameliorated the seizures induced by electric currents or convulsive agents such as pilocarpine, penicillin, and pentylenetetrazole (PTZ) in rodents [12,13,14,15,16,17]. It should be noted that, when CBD is administered before seizure induction, in the acute phase, it reduces seizure severity, but there is little information about how CBD affects the processes that take place during the chronic phase or how it might modify the course of the disease [18,19]. It is important to consider that CBD has anti-inflammatory, antiapoptotic, and neuroprotective effects, possibly due to the existence of cannabinoid receptors both in glial cells and on the surface of B, NK, and T lymphocytes, as all of these cells are involved in neuroinflammation. Several studies confirmed that neuroinflammatory processes may play an important role in epileptogenesis, seizure worsening, or developing epilepsy-associated neuropsychiatric comorbidities (e.g., anxiety, depression, cognitive impairment) [20,21,22,23,24,25,26].

Epileptogenesis can be triggered by lesions of varying duration and intensity that, after a certain period of time, will cause spontaneous and recurrent seizures [27]. Between the initial insult and the emergence of spontaneous seizures, some adaptive changes occur at both the cellular and molecular levels, but these have not yet been fully elucidated [28,29]. It is known that pentylenetetrazole (PTZ) can cause acute seizures in rats at higher doses, but when used at lower, subconvulsive doses, it can produce a chronic epileptic state characterized by a progressive decrease in the seizure threshold and a continuous increase in seizure severity [30]. Behavioral, biochemical, and structural changes in neural development can all be induced by PTZ kindling. Despite lacking spontaneous seizures, a seizure threshold decrease in kindled animals mimics epilepsy phenotypes through an imbalance between the excitatory and inhibitory neurotransmission systems. [31,32,33]. In addition, the PTZ-kindling model has been shown to reflect the cognitive impairments [34,35] and the characteristic cellular changes related to epilepsy in rats, such as astrogliosis [18] and microglia activation [36].

This study aimed to evaluate the effects of chronic CBD administration in the PTZ-kindling model of epilepsy in rats using behavioral tests, bioanalytical assay for brain exposure quantification, and immunohistochemistry to assess cellular alterations, and finally, to evaluate the role CBD plays in cognitive performance change.

## 2. Materials and Methods

### 2.1. Animals

Experimentally naive, adult male Wistar rats were provided by the Biobase of the George Emil Palade University of Medicine, Pharmacy, Science, and Technology of Targu Mures. Before the experiments, all animals were subjected to a 7-day habituation period, when acclimatization to single housing, daily handling, and standard environmental conditions (12 h light–dark cycle, 20 ± 2 °C temperature, 60% ± 10% humidity) were carried out. Standard rodent pellet chow (“Cantacuzino” National Institute of Research and Development) and tap water were provided ad libitum. Body weight was recorded once weekly, and their health status and well-being were monitored daily. The applied procedures were in accordance with European Directive 2010/63/EU and approved by the Ethics Committee for Scientific Research of the George Emil Palade University of Medicine, Pharmacy, Science, and Technology of Targu Mures (approval no. 63/2018).

This study was designed to evaluate the long-term effects of CBD in the PTZ-kindling model of epilepsy. Therefore, to study the protective effects of CBD (60 mg/kg, oral), the drug was administered either 24 h before or 1 h after PTZ injections. The PTZ-kindled animals were randomly divided into two groups, a control group (orally received the vehicle, 1 mL/kg, *n* = 12) and a CBD-treated group (orally administered CBD, 60 mg/kg, *n* = 12). All 24 animals underwent the PTZ-kindling procedure, i.e., intraperitoneal injection of a subconvulsive dose of 35 mg/kg PTZ every other day for 50 days. Both the control and the CBD-treated group underwent the same care and injection protocol, and they were evaluated equally. The third group of animals (sham, *n* = 8) was used to compare the eventual cellular alterations observed by immunohistochemistry; these animals were, every other day, administered i.p. injections of the vehicle instead of PTZ, and they were not treated with CBD.

### 2.2. Drugs and Reagents

**Figure 1:**
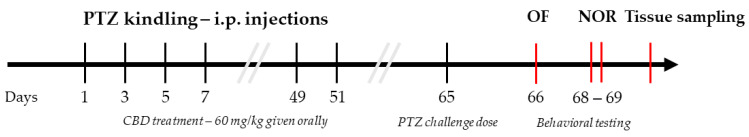
Timeline illustration of the experimental model. Cannabidiol was administered orally in a dose of 60 mg/kg each day, starting from day 0 until achieving a kindled state. Abbreviations: OF, open field test; NOR, novel object recognition test.

Crystalline cannabidiol (99.5% purity from Trigal Pharma GmbH, Wien, Austria), dissolved in extra virgin olive oil (Salov S.p.A., Massarosa, Italy) was administered to the animals. The individually calculated CBD dose based on the previously measured body weight (60 mg/kg body weight) was administered daily by adsorbing CBD oil onto food pellets. Pellets that were not loaded with CBD, but which were coated with olive oil, were administered to the control group. CBD treatment was initiated at day 0, before the first PTZ injection. The dose of CBD was chosen based on previously published results showing that doses below 50 mg/kg body weight did not exhibit anticonvulsant effects [18,37]. An overview of the experimental design is illustrated in Figure 1. 

The plasma concentration and brain penetration ratio of the formulation (i.e., CBD dissolved in virgin olive oil and adsorbed on food pellets) used in this study was determined using plasma and brain samples obtained from anesthetized animals (ketamine–xylazine 100 mg/kgbw and 10 mg/kgbw, respectively) at two preliminarily determined time points (1 h and 24 h) after administration, corresponding to Cmax and Cmin, respectively. Each interval group consisted of 5 animals. Serum and brains were collected, frozen, and kept at −20 °C until analysis. Ketamin hydrochloride (Bela-Pharm GmbH & Co. KG, Vechta, Germany) and xylazine hydrochloride (Bioveta, Ivanovice na Hané, Czech Republic) were used for anesthesia.

### 2.3. Determination of Plasma and Brain Levels of CBD

To ensure the proper selectivity accompanied with high sensitivity for the quantification of CBD in rat plasma and homogenized brain tissue samples, liquid chromatography, coupled with mass spectrometric detection (LC–MS), was used. The system was an Agilent 1100 chromatograph equipped with an Agilent Triple Quadrupole MS detector (Agilent G6410A1, Technologies, Santa Clara, CA, USA), realizing the separation by using a Kinetex Polar C18 (100 × 4.6 mm, 2.6 μm, Phenomenex, Torrance, CA, USA) stationary phase with a flowrate of 0.5 mL/min, at 30 °C, with a total runtime of 4.5 min. To support specificity, negative electrospray ionization with multiple-reaction monitoring (MRM) mode was applied, quantifying the ion with 245 *m*/*z*, derived from 313 *m*/*z*. The potential fragmentation pathway of cannabidiol and the characteristic MRM mass spectrum are presented on Supplementary Figure S1, while the detailed chromatographic conditions are summarized in Supplementary Table S1. The method was partially validated in accordance with the ICH Q2(R2) guideline, demonstrating the specificity, linearity, accuracy, and limit of quantification. The results of the validation procedure are presented in Supplementary Table S2. The obtained chromatograms for the limit of quantification, and representative chromatograms of the plasma and homogenized brain tissue samples are presented on Supplementary Figure S2.

Methanol (Sigma-Aldrich, Steinheim, Germany), acetonitrile (Merck, Darmstadt, Germany), formic acid (Scharlau Chemie, Sentmenat, Spain), and water (Millipore Direct Q10, Merck Millipore, Burlington, MA, USA) used for analytical procedures were of HPLC grade.

Brain and plasma samples were collected on the last day of the experiment (day 70 and day 71, respectively) and underwent the same processing steps as described previously [38]. Briefly, blood obtained via cardiac puncture in K3 EDTA tubes was centrifuged at 3000 *g* for 10 min at 4 °C within 2 h of collection. The plasma samples were frozen at −20 °C until further pre-analytical processing. Following blood collection, the animals were perfused with approximately 30 mL of normal saline until the entire volume of blood was removed. This technique ensures that the measured concentrations reflect the concentrations in brain tissue by eliminating contributions from brain vasculature. The brains were removed within 7 min of when the thorax was opened. Tissue samples were weighed, homogenized in 5 mL phosphate buffer (PBS) in a grinding ball mill (UltraTurrax Tube Drive, IKA, Königswinter, Germany) for 10 min, and stored at −70 °C until analysis. 

Plasma samples were diluted with blank plasma if necessary and mixed with 3 volumes of methanol to induce plasma protein precipitation. The mixture was vortexed for 10 s and then centrifuged (Sigma 2–15 centrifuge, Sigma, Osterode am Harz, Germany) for 10 min at 9167 g. The supernatant was diluted with a mobile phase and injected into the LC-MS/MS system. Homogenized brain tissue samples underwent the same procedures as plasma samples.

### 2.4. Pentylenetetrazole Induced Kindling Model (PTZ-Kindling)

Pentylenetetrazole (PTZ, Sigma Aldrich, St. Louis, MO, USA) was dissolved in 0.9% saline at a concentration of 35 mg/mL and injected intraperitoneally (i.p.) in a volume of 1.0 mL/kg at a sub-convulsive dose of 35 mg/kg according to the previously published schedule [39,40]. Seizure scoring was performed in real-time, followed by two blinded observers’ confirmation of the registered data. Briefly, after each PTZ injection, rats were housed singly in transparent plexiglass cages and monitored for 1 h. Seizure intensities were rated by one experienced observer according to a modified Racine scale as follows: 0 = no response; 1 = ear and facial twitching; 2 = myoclonic jerks without rearing; 3 = myoclonic jerks with rearing; 4 = turning over into side position with tonic-clonic seizures; 5 = turning over into back position, generalized tonic-clonic convulsions, and loss of balance and falling. The same scoring scale was used by the two blinded observers who analyzed the video registrations offline. The final seizure score was established by combining the scores given by each observer.

An animal was considered kindled when it had experienced stage 4 or 5 seizures on two consecutive trials. At the beginning of the experiment, the sensitivity to the convulsant action of PTZ was assessed, and animals having two consecutive stage 5 seizures after the first two PTZ doses were not included in the study.

### 2.5. Behavioral Assays

For the assessment of the behavioral aspects, open field (OF) and novel object recognition (NOR) tests were performed at days 66 and 68–69, respectively (Figure 1).

#### 2.5.1. Open Field Test

The OF test was performed in a 60 × 60 cm black-based box with transparent walls, with a height of 50 cm, to observe the animals’ locomotor and exploratory behavior. The illumination in the testing room was controlled and maintained between 50 and 100 lux. After the animals were placed in the middle of the testing area, their behavior was recorded for five minutes from above. After each test, 70% ethanol was used to disinfect the apparatus. All trials were analyzed offline with EthoVision XT (version 11.5, Noldus IT, Wageningen, The Netherlands), monitoring the distance moved, the number of entries, and the time spent in the center zone (central 30 × 30 cm area), vertical activity (wall climbing, rearing), and grooming activity.

#### 2.5.2. Novel Object Recognition Test

In an empty testing chamber (60 × 60 cm), animals were habituated for 10 min, then returned to their home cage, and the chamber was cleaned as described above. In the familiarization phase, two identical novel objects constructed of wood (10 × 4 × 4 cm) were placed in the chamber (30 cm apart from each other) (Supplementary Figure S3). Object placement was chosen so that animals could walk freely around the arena’s edge, as they would have done in an open field. Thus, the animals were required to move into the center of the chamber to interact with the objects rather than unintentionally encountering them as they explored the arena perimeter.

Observations of animal activity were conducted for 5 min. A nose touch was classified as an interaction with an object. For the subsequent memory test, the animals had to interact with each object for at least 2.5 s during the familiarization phase. The animals were returned to their home cages after their five-minute time frame had ended. Before each test, the chamber and objects were cleaned with a 70% ethanol solution. 

The inter-trial interval between familiarization and testing was 24 h. One of the familiar objects encountered during the familiarization phase was then replaced with a novel object, which was different in color and configuration but about the same size. Across tests, the position of the novel object (i.e., left or right) was counterbalanced. Following the same procedure as the one during familiarization, animals were reintroduced to the chamber and allowed to explore it for five minutes. Time spent by the animal in exploring individual objects during familiarization phase, total time spent by the animal in exploring both objects during the test and training phase, the total distance moved, the time spent moving, and the number of rears were quantified by a computerized analysis system (EthoVision XT, version 11.5, Noldus IT, Wageningen, The Netherlands). 

### 2.6. Histological Staining

#### 2.6.1. Perfusion and Brain Sectioning

A mixture of ketamine–xylazine (100 mg/kgbw and 10 mg /kgbw) was injected intraperitoneally to induce deep anesthesia. Rats were perfused transcardially with ice-cold normal saline solution (0.9%, for 1.5 min), followed by ice-cold fixative solution containing 4% paraformaldehyde (Sigma Aldrich, St. Louis, MO, USA) and 0.25% picric acid (Sigma Aldrich, St. Louis, MO, USA) in 0.1 M phosphate buffer (PB, pH = 7.4, Sigma Aldrich, St. Louis, MO, USA) for 20 min. After perfusion, the brains were removed from the skull and postfixed overnight in 4% PFA. Then, 60 μm-thick coronal sections were cut with a vibratome (VT 1000S, Leica, Nussloch, Germany) and washed in 0.1 M PB.

#### 2.6.2. Fluorescent Immunohistochemistry

Triple immunofluorescent staining was used to visualize neurons, astrocytes, and microglia. Brain sections were transferred into a 24-well tissue culture plate (TPP, Trasadingen, Switzerland) and were immunostained in a free-floating manner in a 500 μL volume on an orbital shaker (Heidolph Instruments, Schwabach, Germany). After three 10 min washes in 0.1 M PB, sections were washed three times for 10 min in tris-buffered saline (TBS), then blocked in TBS containing 10% normal horse serum (NHS; Vector Laboratories, Burlingame, CA, USA) and 0.1% Triton-X (Sigma-Aldrich, St. Louis, MO, USA) for 45 min, in order to block nonspecific binding sites and to enhance antibody penetration.

Sections were then incubated with the primary antibodies against NeuN for neurons (NeuN; guinea pig raised-polyclonal, dilution 1:500; product no: 266004, Synaptics Systems GmbH, Goettingen, Germany), GFAP for astrocytes (GFAP; mouse raised-monoclonal dilution 1:500; product no: 173211, Synaptics Systems GmbH, Goettingen, Germany), and IBA1 for microglia (IBA1; rabbit raised-polyclonal, dilution 1:500; HistoSure: HS234013, Synaptics Systems GmbH, Goettingen, Germany) in 0.1% TBS-T overnight at room temperature.

The next day, sections were washed thoroughly in TBS, then fluorescently labeled secondary antibodies made up in TBS were applied at room temperature for 4 h to label the NeuN immunostaining with Alexa488-conjugated donkey anti-guineapig (1:500, Jackson ImmunoResearch Laboratories, West Grove, PA, USA), to label the GFAP immunostaining with Alexa647-conjugated donkey anti-mouse (1:500, Jackson ImmunoResearch Laboratories, West Grove, PA, USA) and to label the IBA1 immunostaining with Alexa594-conjugated donkey anti-rabbit (1:500, Jackson ImmunoResearch Laboratories, West Grove, PA, USA).

Afterwards, stained sections were washed 3 times for 20 min in TBS and 2 times for 10 min in 0.1 M PB, mounted on slides and coverslipped with a mounting medium (Vectashield, Vector Laboratories, Burlingame, CA, USA) and sealed with nail polish. 

#### 2.6.3. Confocal Image Acquisition and Analysis of Fluorescent Immunostaining

The digitalization of sections was conducted using confocal microscopy. To obtain high-resolution z-stacks, all images were acquired using a Leica TCS SP8 confocal laser scanning microscope (Leica Microsystems GmbH, Wetzlar, Germany) using HC PL APO CS2 20X/0.75 and HC PL APO CS40X/0.85 dry objectives and unidirectional scanning at 200 Hz. Images were processed and quantified using the Leica Application Suite X (Leica Microsystems GmbH, Wetzlar, Germany) software. High-magnification images of 1024 × 1024 pixels were collected, and regions of interest (ROIs) in individual sections were selected (400 μm × 600 μm). Z-stack deepness was defined as 5 μm, each image comprising of 3 subsequent z-stack layers, resulting in 10 μm-deep recordings. The image of the hippocampal sample was acquired in three different channels. The NeuN staining was used to distinguish hippocampal regions and layers based on the density and relative location of the cells. Light signals from photon scatter around the edges of tissue, tears in the tissue, and vasculature were excluded from analysis. 

Fluorescence intensity was measured over ROIs, then corrected for autofluorescence and non-specific signals using a background subtraction. Astrocytes, microglia, and neuronal debris in CA1 and CA3 were consistently counted in the same area in all slices and were expressed as cells/mm^2^. Image J software was used for manual cell-counting. Three sections from each slide, four slides per animal, and five to eight animals per group were used for histological assessment. 

##### Custom Cell Counter Algorithm

**Figure 2:**
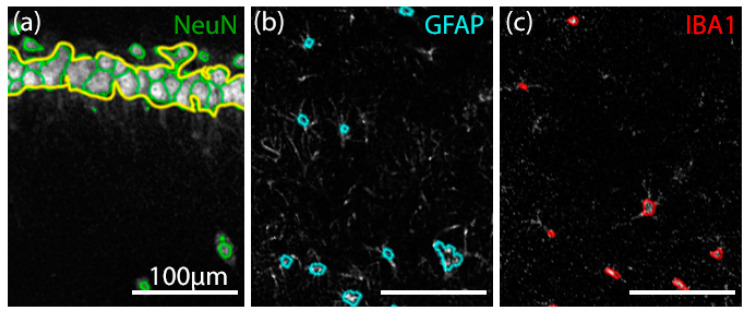
Analysis of different cell types by a cell-counter algorithm. (**a**) NeuN+ cells (green) with an explicitly delimited pyramidal layer (yellow), (**b**) GFAP+ (cyan), and (**c**) IBA1+ (red) cells evidentiated and contoured by our custom cell-counter algorithm. All three immunostainings are superposed in their z-stack maximum-intensity projections.

For the validation of cell-counting procedures, all ROIs previously investigated were cropped and split to separate RGB (red–green–blue) channels using the Image J software. Each immunostaining channel (green for NeuN, cyan for GFAP, and red for IBA1, respectively) were further analyzed by our custom cell-counter algorithm written in the Python programming language (version 3.9.9), implementing the OpenCV library (version 4.5.5). ROIs were preprocessed by Gaussian blurring, in order to reduce background noise. Desirable foreground image objects were evidentiated using adaptive thresholding, which was followed by morphological opening and closing functions, based on the extent of cell clustering and overall image quality. Next, the area and longest diagonal of all cell-like structures were calculated. If any surface detected had an overlap of at least 60% with another in the subsequent layers and its longest diagonal exceeded the value corresponding to 6.5 μm, the element could be considered a cell. Cellular debris was defined as NeuN-positive fragments with dimensions between 2.5 and 6.5 μm, encircled by GFAP-positive astrocyte signals as described previously [41]. Finally, all cell contours were projected on the original ROI, thus enabling a visual inspection of the detected structures (Figure 2).

### 2.7. Statistical Analysis

Data were analyzed using GraphPad Prism 8 (version 8.0.1, GraphPad Software Inc., San Diego, CA, USA). Differences in survival curves were tested using the log-rank test (Mantel-Cox). Kolmogorov–Smirnov tests were performed on each set of data to determine whether it had a normal distribution. Non-normally distributed data was analyzed with a Mann–Whitney test; otherwise, an unpaired t test was used. For matched observations, a two-way ANOVA with Sidak’s multiple comparison test was used. The mixed-effect model with Geisser–Greenhouse correction was used in case of missing values. An alpha value of 0.05 was used as the cutoff for significance. 

## 3. Results

### 3.1. Seizure Score

**Figure 3:**
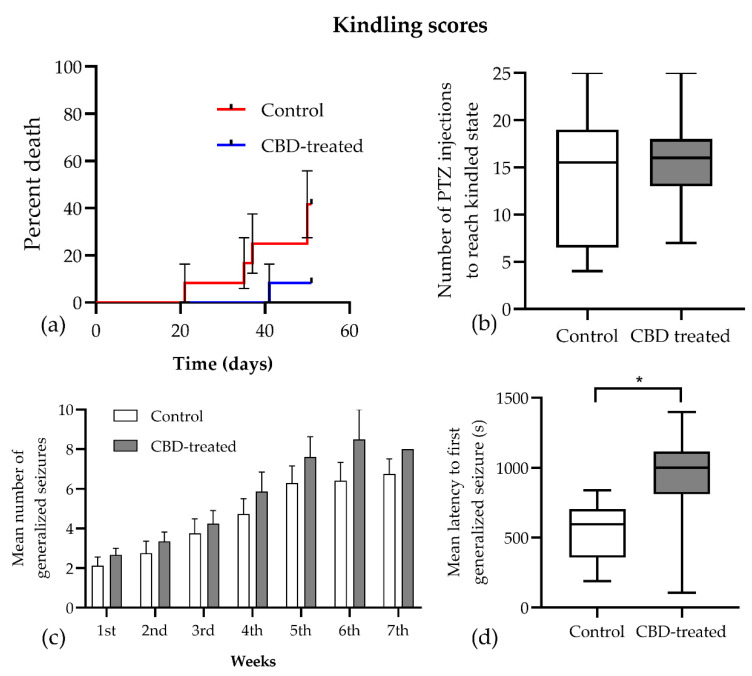
Effects of chronic CBD treatment on the kindling scores induced by pentylenetetrazole in rats. The development of kindling was characterized by the following parameters: (**a**) mortality rate due to generalized seizures, (**b**) the total number of PTZ injections to reach the kindled state, (**c**) the number of generalized seizures, and (**d**) the latency to the first generalized seizure. Data are expressed as mean ± SEM in the bar graph and as mean (solid line) with the range in the floating bar graphs; * *p* < 0.05 vs. control.

Twenty-four rats were used for PTZ kindling, from which 18 ended up reaching a kindled state and, due to this, were each consecutively tested with a challenge dose of PTZ. The remaining six animals (*n* = 5 in the kindled and *n* = 1 in the CBD-treated group) died before completing the study, so the behavioral assessment was performed on *n* = 7 kindled and *n* = 11 CBD-treated animals. The mortality rate tended to decrease following chronic CBD treatment (5/12 vs. 1/12, Chi square = 3.429, *p* = 0.064, Figure 3a), especially in the early phase of the kindling. 

Analyzing the parameters characterizing the development of the kindling process, it was observed that the total number of PTZ injections required to achieve a kindled state did not show a significant difference between CBD-treated and control groups (median 15.5 vs. 16, *p* = 0.833, Figure 3b). The seizure scores registered during kindling were analyzed using the mixed-effect model with Geisser–Greenhouse correction, and it was noted that the time factor was significant in the development of generalized seizures (Racine 4 and 5 seizures) for both groups (F (1.667, 16.39) = 53.63, *p* < 0.001, Figure 3c). However, treatment and the time x treatment interaction were not significant (F (1, 15) = 2.397, *p* = 0.142 and F (6, 59) = 1.174, *p* = 0.333, respectively). Furthermore, there was no difference in the duration of generalized seizures between the CBD-treated and control groups (74 ± 25.7 vs. 89 ± 40 s, *p* = 0.425). Conversely, the mean (± SEM) latency to first generalized seizure was significantly longer in the CBD-treated group (925.3 ± 120.0 vs. 550.1 ± 69.62 s, Figure 3d), and the mixed-effects analysis confirmed that treatment had a significant influence on this parameter (F (1, 15) = 6.3872, *p* = 0.023).

**Figure 4:**
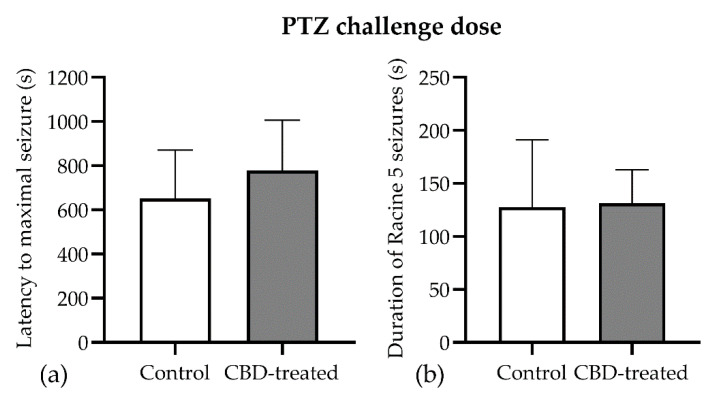
Lack of long-term effect of CBD (60 mg/kg) in the PTZ-kindling model. (**a**) Latency to maximal seizure observed after PTZ injection. (**b**) Duration of the generalized seizures (clonic convulsion with loss of righting reflex and/or bouncing, two or more clonic convulsions, tonic convulsion or status epilepticus) induced by a challenge dose of PTZ. Data are expressed as mean ± SEM (*n* = 7–11) for each parameter.

The challenge dose of PTZ induced the same seizure pattern in both groups, and all animals had Racine 5 seizures that did not differ in duration between groups (127.4 ± 63.58 vs. 131.2 ± 31.53 s, *p* = 0.312). However, the latency to maximal seizure in the CBD-treated group showed high variability among animals (777.8 ± 228.4 vs. 651.1 ± 220.0 s, Figure 4a) with no significant differences between groups (t(15) = 0.397, *p* = 0.697).

### 3.2. CBD Plasma and Brain Concentrations

To verify whether the in-house-prepared CBD solution administered by the oral route would achieve the desired concentrations at the site of action, serum and brain CBD concentrations were assessed at 1 h and 24 h after administration, corresponding to Cmax and Cmin, respectively. The mean peak concentration was 1976.1 ± 1151.41 ng/mL in serum and 5260 ± 3284 ng/g in brain, while the minimum concentrations were 24.2 ± 3.25 ng/mL and 91.4 ± 20 ng/g, respectively (Table 1). Brain-to-plasma ratios calculated for each animal showed higher values at 24 h compared to 1 h after administration, suggesting a slower brain CBD-elimination rate.

### 3.3. Open Field Test

**Figure 5:**
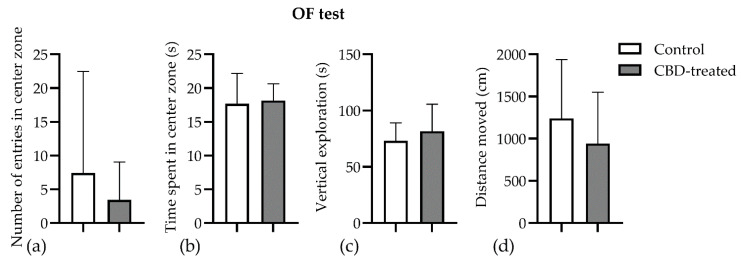
Assessment of locomotor activity in the open field. Rats were submitted to a PTZ-kindling protocol with or without chronic cannabidiol (60 mg/kg body weight) administration (mean ± SEM, *n* = 18). (**a**) The number of entries in the center zone; (**b**) time spent in the central zone of the arena, defined as a 30 × 30 cm square out of an 60 × 60 cm total surface of the arena; (**c**) the vertical exploration of the animals was expressed as the time spent leaning on the walls of the arena; (**d**) total distance moved during the 5 min testing session. CBD, cannabidiol. Data are expressed as mean ± SEM (*n* = 7–11) for each parameter.

Chronic CBD treatment had no effect on exploratory and locomotor parameters in the open field test. The control animals apparently entered more times in the center zone of the arena (7.4 ± 15.1 vs. 3.43 ± 5.62, *p* = 0.77, Figure 5a), but they spent the same amount of time exploring it (17.69 ± 4.48 vs. 18.15 ± 2.49, *p* = 0.92, Figure 5b). The distance travelled and the vertical exploration expressed as supported rearings did not vary between groups (*p* > 0.05) (Figure 5c,d).

### 3.4. Novel Object Recognition Test

**Figure 6:**
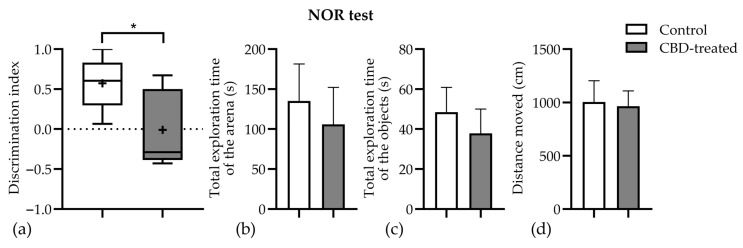
The effects of chronic CBD (60 mg/kg body weight) treatment on cognitive performance of rats subjected to a PTZ-kindling protocol. (**a**) Discrimination index (DI), which shows the discrimination between the novel and familiar objects, i.e., the difference in exploration time for a familiar object, but then dividing this value by the total amount of exploration of the novel and familiar objects [DI = (TN − TF)/(TN + TF)] showed a significant decrease in the CBD-treated group. + sign shows the arithmetic mean, whereas the line represents the median; (**b**) the total exploration time of the arena (mean ± SEM) did not show statistically significant difference between groups; (**c**) the total exploration time of the objects (mean ± SEM) was also similar for both groups; (**d**) the total distance moved by both groups (mean ± SEM) during the 5 min testing session was almost equal, and the results were similar to those found in the OF test. CBD, cannabidiol; * *p* < 0.05 vs. control. Data are expressed as mean ± SEM (*n* = 7–11) for each parameter.

There was no effect of CBD treatment on the total distance traveled and on the exploration time of the arena or objects. Conversely, the mean discrimination index was decreased by CBD treatment (0.57 ± 0.15 vs. −0.01 ± 0.17, *p* = 0.0367, Figure 6). The results indicate that CBD-treated animals were impaired in distinguishing between the novel and familiar objects.

### 3.5. Fluorescent Immunohistochemistry

#### 3.5.1. Validation of Manual and Algorithmic Cell Counter Strategies

**Figure 7:**
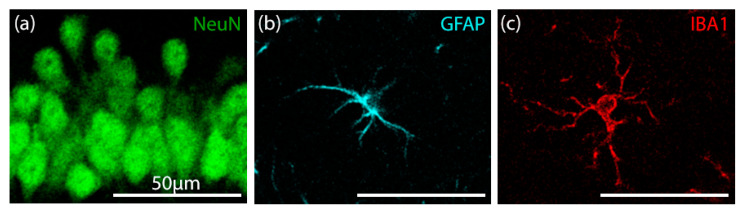
Neuron, astocyte, and microglia triade. Representative confocal microscopy image of the tripple immunostaining of the (**a**) neuron-NeuN, (**b**) astrocyte-GFAP, and (**c**) microglia-IBA1 in the CA3 subfield of the hippocampus.

Neurons, astrocytes, and microglia were triple-immunostained in the hippocampus, where the CA1 and CA3 regions were analyzed (Figure 7). Sham, i.e., healthy control (*n* = 6), PTZ-kindled control (*n* = 5), and CBD-treated (*n* = 8) groups showed different pattern of immunostaining for astrocytes labeled with anti-GFAP antibody (cyan), for neurons labeled with anti-NeuN antibody (green), and for microglia labeled with anti-IBA1 antibody (red). 

We used a custom cell-counter algorithm to quantify the cells of interest. The manual counting method was used as the reference method. The mean of the two observers was compared with the values provided by the algorithm. For the statistical analysis the bias, precision and limits of agreement were used as proposed by Bland and Altman to compare two methods of measurement. The methods were considered to be interchangeable if the bias was smaller than ±10% for each cell type and if there was no tendency of the difference to increase with the mean (Supplementary Figure S4).

#### 3.5.2. Effects of CBD on the Neuron–Astrocyte–Microglia Triad in the Hippocampus

**Figure 8:**
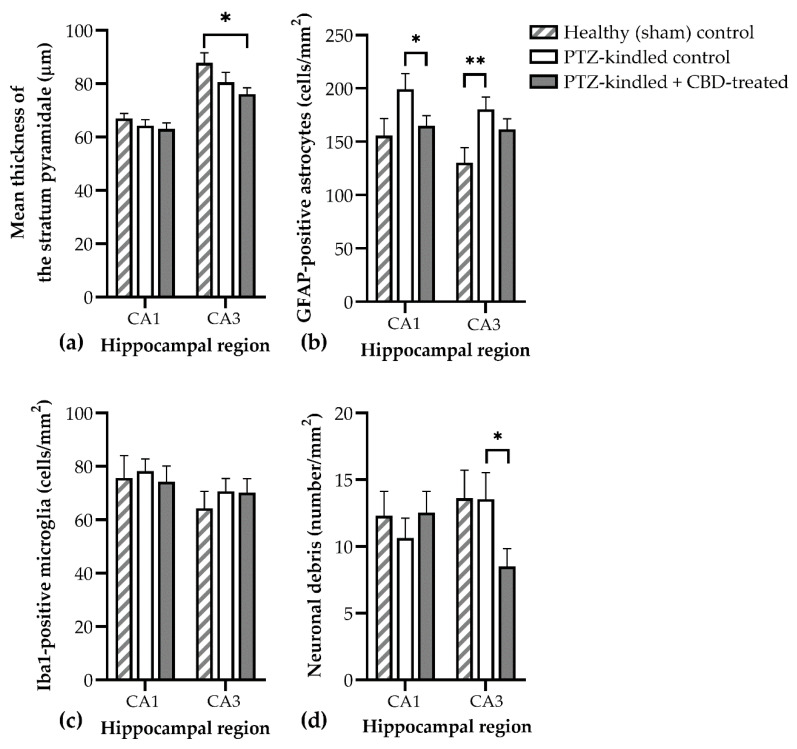
Effects of chronic CBD (60 mg/kg body weight) treatment on the hippocampal reorganization induced by PTZ-kindling in rats. (**a**) Mean thickness of the pyramidal cell layer; (**b**) the density of GFAP-positive astrocytes; (**c**) the density of IBA1-positive microglia; (**d**) the density of neuronal debris, defined as NeuN-positive fragments surrounded by astrocytes. Data are expressed as mean ± SEM (*n* = 5–8).; CBD, cannabidiol; * *p* < 0.05; ** *p* < 0.01.

The thickness of the stratum pyramidale showed differences between groups, a two-way ANOVA analysis showing a significant impact of CBD treatment (F (2, 119) = 3.963, *p* = 0.02). The PTZ-kindling procedure decreased the stratum pyramidale thickness compared to sham animals, and CBD treatment induced a further significant thinning in the CA3. A similar tendency could be observed in the pyramidal layer of the CA1 region as well, but it did not reach significance (Figure 8a, Supplementary Figure S5). 

The cell density of GFAP-positive astrocytes was decreased by CBD treatment (F (2, 119) = 6.546, *p* = 0.002,) both in the CA1 and CA3 regions of the hippocampus (Figure 8b). The post hoc analysis confirmed that the CBD-treated group had significantly decreased astrocyte density in the CA1 region (*p* = 0.045). Conversely, neither the PTZ-kindling nor the CBD treatment had any effect on the total number of IBA1-positive cells (F (2, 119) = 0.2522, *p* = 0.78, Figure 8c).

Neuronal debris, i.e., fragments of NeuN positive cells closely attached to the branches of astrocytes in the stratum radiatum, was observed in both the CA1 and the CA3 regions. Despite the fact that PTZ-kindling did not influence the density of neuronal debris when compared to sham animals, the CBD-treated group had significantly decreased neuronal debris in the CA3 region of the hippocampus when compared to PTZ-kindled controls (Figure 8d, *p* = 0.0359).

## 4. Discussion

This study revealed that chronic treatment with CBD reduced seizure-related mortality and prolonged the latency for the appearance of generalized seizures in PTZ-kindled rats. However, the development of kindling and the maximum seizure severity were not influenced. Interestingly, CBD treatment decreased the cognitive performance of rats in the NOR test. Furthermore, at the cellular level, the CA3 and CA1 regions of the hippocampal formation of CBD-treated rats showed significant differences compared to PTZ-kindled controls, which may explain the decreased mortality and cognitive performance. 

CBD has been demonstrated to affect the central nervous system dose-dependently without any psychoactive action. In low doses, it showed anxiolytic and antidepressive-like effects [4,42,43,44,45,46,47] like serotonergic drugs [48,49], whereas in higher doses (30–100 mg/kg), its anticonvulsant and antipsychotic actions were proven [1,16,18,19,37]. There are, however, many discrepancies between the previously reported results, which may partly be attributed to the unfavorable pharmacokinetic properties of CBD. It is possible that highly lipophilic drugs such as CBD can precipitate in the stomach when administered orally in the form of suspensions, resulting in prolonged absorbtion, a long time to peak plasma concentration, and low bioavailability [50]. Moreover, the vehicle of administration was shown to significantly influence the Cmax and AUC of CBD [51]. Although this issue is clearly present in laboratory experiments as well, very few studies have provided data about the pharmacokinetics of CBD formulations administered to animals [37,52].

To achieve better bioavailability, CBD was dispersed in olive oil and incorporated into the food pellets. The obtained results showed that brain concentrations correlated well with the plasma concentrations, and the brain-to-plasma ratio was consistently higher 24 h after administration, which indicates the high affinity of CBD to brain tissue. These brain concentrations are very similar to those reported recently by Uttl et al. [37]. However, plasma concentrations obtained at 1 h after administration showed that, in some cases, absorbtion was not complete. So, this study confirmed that CBD, even when dissolved in oil, which is the most common formulation of CBD used in humans, showed significant variations in the absorption phase, making the study of peak-time effects challenging, which could underlie the controversial results published previously. Hence, future studies should relate CBD’s effects to its plasma concentration, rather than the dose administered.

The most important finding of this study is that CBD decreased seizure-related mortality and increased the latency to tonic-clonic seizures in the PTZ-kindling model, although it was not tested at peak plasma/brain concentrations. From a translational point-of-view, this result may provide evidence for the long-term protective action of CBD against the progression of epileptic syndromes and the potential prevention of breakthrough seizures. As previously reported, a single administration of CBD at different doses exhibits anticonvulsant effects in several animal models of epilepsy, but it does not necessarily reduce the frequency or the severity of seizures. In contrast, CBD modified the duration of generalized seizures, the latency to seizure onset, and the mortality [1,12,14,16,18,19,53]. In pilocarpine-induced seizures, CBD administered at 100 mg/kg doses did not affect the severity of seizures [14]. However, the same study reported a reduction in mortality and the occurrence of tonic-clonic seizures in the penicillin model of partial seizure [14]. The reduction of seizure duration and the increase of the latency to first seizure by CBD at a dose of 60 mg/kg were also reported after the acute intraperitoneal administration of PTZ [19]. 

When administered for a longer period, CBD delayed the progression of kindling in PTZ-kindled mice, but it did not prevent generalized seizures [19]. In rats, Mao et al. reported a decrease of the mean seizure score and the kindling rate in the CBD-treated group (50 mg/kg administered intraperitoneally) [18]. However, CBD was administered before PTZ injections in both studies; thus, the observed anticonvulsant action corresponds to the peak-time effect of CBD described after acute administration. In an interesting approach, Hosseinzadeh et al. demonstrated that the intracerebroventricular injection of CBD might have long-term protective effects in the pilocarpine model of epilepsy in rats [16], and the neuroprotective effects of CBD described by in vitro and in vivo studies [1,54] point to the potential benefits of chronic adjunctive treatment with CBD. On the other hand, it is important to differentiate between epileptic seizures and epilepsy syndromes. CBD is currently approved for the treatment of seizures associated with Lennox–Gastaut syndrome or Dravet syndrome based on a favorable benefit–risk profile [10], but there is little evidence about its effectiveness in other types of epileptic seizures. Therefore, the present study proposed to study the effects of CBD on seizures by delivering the seizure-precipitating factor (i.e., PTZ injection) outside of CBD’s maximal anticonvulsant protection. The only significant difference between the treatment groups was the prolonged latency to the first generalized seizure; the other parameters related to kindling were not modified by CBD treatment. Interestingly, this led to a reduced mortality rate in the CBD-treated group, which is in agreement with previous studies [12]. 

The next important finding of this study is the effects of chronic CBD treatment on the cognitive performance of PTZ-kindled rats. CBD has been proposed to exert neuroprotective effects in epilepsy [55] and possesses several mechanisms to protect against memory impairments in various diseases [23]. However, a dose-dependent nature of these neuroprotective effects was also demonstrated, the lower and middle range doses (5–20 mg/kg) showing improvements in memory assessment tasks [56]. In this experiment, the decrease of the discrimination index in the NOR test clearly showed that CBD-treated animals could not recall the familiar object and interacted with both familiar and novel objects more equally. Studies have shown that the hippocampus plays a significant role in object-recognition memory. If this structure is damaged, there will be moderate and reliable changes in anterograde memory [57]. The histological analysis of the hippocampal formation revealed that CBD-treated animals had a reduced thickness of the stratum pyramidale in the CA3 and CA1 regions. As confirmed by others, this measurement of stratum pyramidale thickness is robust and constant, because it does not differ across a series of coronal sections collected around the anteroposterior axis of the same animal [58]. Additionally, stratum pyramidale thickness was shown to reflect the neurodegeneration caused by toxic substances such as cadmium [59] and amyloidbetapeptide 1–40 [60] in rat models with cognitive impairments similar to those observed in this study.

On the other hand, CBD treatment decreased the number of GFAP-positive astrocytes to sham levels. The results of this study confirmed again that PTZ-kindling induces astrogliosis in the CA3 and CA1 regions of the hippocampus [61] and that CBD treatment prevents the proliferation of astrocytes [18]. Astrocytes were demonstrated to play an important role in epileptogenesis by releasing inflammatory cytokines and altering the excitability of the neurons [62]. The reduction in mortality in the CBD-treated group may be due to the long-term antiproliferative effects of CBD on astrocytes, which may be part of a promising novel therapeutic strategy targeting neuroinflammation [63]. 

Furthermore, it is important to note that the number of microglial cells in the hippocampus was not significantly influenced by CBD; however, a decreasing trend was observed. Microgliosis was also linked with epileptogenesis, especially in the status epilepticus models of epilepsy [64]. Although it seems to play a role in the increased excitability of the neurons, its involvement in cognitive impairment was not demonstrated yet. As a limitation of the study, it should be noted that only one microglia-specific histological marker was used (i.e., IBA1), which did not allow the investigation of microglial activation. Another important cellular marker of neuronal apoptosis may be neuronal debris, as described by Lana et al. [41,65,66,67]. This study showed that CBD-treated rats had a decreased number of neuronal debris in the stratum radiatum, despite a lower number of astrocytes, which suggests that microglial activity had an important contribution to the clearance of neuronal debris in this group. Further studies using specific microglial activation and apoptosis markers are needed to fully understand the effects of CBD on the interplay between neurons, astrocytes, and microglia.

## 5. Conclusions

Chronic CBD treatment did not prevent PTZ-induced kindling in rats, but a reduction in mortality associated with prolonged status epilepticus was observed. Analyzing the seizure activities, it was found that the frequency and duration were not influenced by CBD, but the latency to the first generalized seizure was increased. The NOR test showed that CBD treatment impaired recognition memory, which may warrant further investigation. The main findings of the study (i.e., reduced mortality and cognitive dysfunction) may be connected to the observed hippocampal histological changes, such as reduced thickness of the stratum pyramidale or decreased astrogliosis.

## Figures and Tables

**Table 1:** Cannabidiol concentrations in plasma and brain at Cmax and Cmin after the oral administration of 60 mg/kg body weight.

CBD Concentrations	Rat #	Plasma Concentration (ng/mL)	Brain Concentration (ng/g)	Brain-to-Plasma Ratio
1 h after administration	1	259.93	1058.50	4.072
	2	6326.57	16,886.61	2.669
	3	800.39	1475.58	1.844
	4	198.69	642.07	3.232
	5	2295.02	2455.34	1.070
	Mean	1976.12	5260.6	2.577
	SEM	1151.41	3284.0	0.524
24 h after administration	6	23.20	111.08	4.788
	7	26.20	79.98	3.053
	8	28.67	105.28	3.672
	9	22.50	- *	-
	10	20.40	69.35	3.400
	Mean	24.19	91.4	3.728
	SEM	3.25	20.0	0.336

* As a result of inadequate tissue perfusion, the brain tissue sample was excluded from analysis. # Data points are labeled by rat number.

## Data Availability

The data presented in this study are available on request from the corresponding author.

## Supplementary Materials

The following supporting information can be downloaded at: https://www.mdpi.com/article/10.3390/biomedicines10081811/s1, Figure S1: The potential fragmentation of CBD in negative electrospray ionization and the characteristic MRM mass spectrum of CBD (313 *m*/*z* → 245 *m*/*z*, collision energy: 20 eV); Figure S2: The chromatograms obtained for the quantification limits (A: plasma with 1 ng/mL CBD and B: homogenized brain sample with 1 ng/ml CBD) and the representative chromatograms of the real samples (C: plasma with 47.3 ng/ml CBD and D: homogenized brain sample with 6.0 ng/ml CBD); Figure S3: Schematic illustration of the novel object recognition task; Figure S4: Bland–Altman plots representing the agreement between the cell counter algorithm and human manual counting of NeuN, GFAP, and IBA1 positive cells in the rat hippocampus; Figure S5: Representative image of morphological changes in the CA3 hippocampal pyramidal layer of (a) PTZ kindled controls and (b) CBD treated rats; Table S1: The chromatographic conditions and the MS detector parameters used for the analysis of plasma and homogenized brain tissue samples; Table S2: The summary of the analytical method validation process.
