# Supplementary material for: Anticonvulsant Action and Long-Term Effects of Chronic Cannabidiol Treatment in the Rat Pentylenetetrazole-Kindling Model of Epilepsy
Source: Biomedicines. 2022 Jul 28;10(8):1811. doi: 10.3390/biomedicines10081811 (PMC9405483; doi:10.3390/biomedicines10081811)
Supplement: Supplementary file 1 [file biomedicines-10-01811-s001.zip › biomedicines-1803265-SI.pdf]

Anticonvulsant Action and Long-term Effects of Chronic Cannabidiol Treatment in the Rat Pentylenetetrazole-kindling Model of Epilepsy

Zsolt Gáll [1,†,*], Krisztina Kelemen [2,†], Andrea Tolokán [1], István Zolcseak [1], István Sável [1], Réka Bod [2], Elek Ferencz [3], Szende Vancea[3], Melinda Urkon [1] and Melinda Kolcsár [1]

Supplementary Table S1. The chromatographic conditions and the MS detector parameters used for the analysis of plasma and homogenized brain tissue samples

**Chromatographic conditions**	
System	Agilent 1100 with Triple Quadrupole 6450 MS detector
Column	Kinetex Polar C18, 100 × 4.6 mm, 2.6 µm (Phenomenex)
Column temperature	30 °C
Flow rate	0.5 ml/min
Mobile phase	0.2 % formic acid in water-acetonitrile 15:85
Injection volume	10 µL
Retention time of CBD	2.6 min
Total run time	4.5 min
**MS detector parameters**	
Ionization	Negative electrospray ionization
Detection	MRM (313 *m/z* → 245 *m/z*)
Dry heat	300 °C
Nebulizer gas pressure	40 psi
Voltage	4000 V
Dry gas	Nitrogen at a rate of 8L/min
Collision energy	20 eV

Supplementary Table S2. The summary of the analytical method validation process

Performance criteria	
Specificity	Selectivity of the method was proved by the injection of biological matrix samples (plasma and brain) and no disturbing peaks were detected at the retention time of cannabidiol. In addition, the use of MRM mode increases the specificity of the determination.
Linearity	To demonstrate the linearity of the method, cannabidiol stock solution was prepared and added to plasma and homogenized brain tissue samples at six levels in the range of 1-50 ng/mL. Calibration curves were represented by plotting peak areas against corresponding concentrations (expressed in ng/mL). The correlation coefficients were determined by linear least squares regression analysis resulting 0.98 for plasma and 0.99 for homogenized brain tissue samples.

Accuracy	The accuracy of the method was tested on six different levels covering the linearity range. The accuracy samples were prepared by adding known amounts of cannabidiol stock solution to plasma and homogenized brain samples and the results were evaluated based on the recovery % values. The mean recovery in the case of the plasma samples was 100.02 % and for the homogenized brain samples was 99.98 %.
Limit of quantification (LOQ)	LOQ values were determined separately for plasma and homogenized brain samples based on signal-to-noise ratios of 10:1. In both cases the LOQ was 1 ng/ml, thereby in case of 10 µL injection volume the absolute sensitivity of the method is 1 pg.

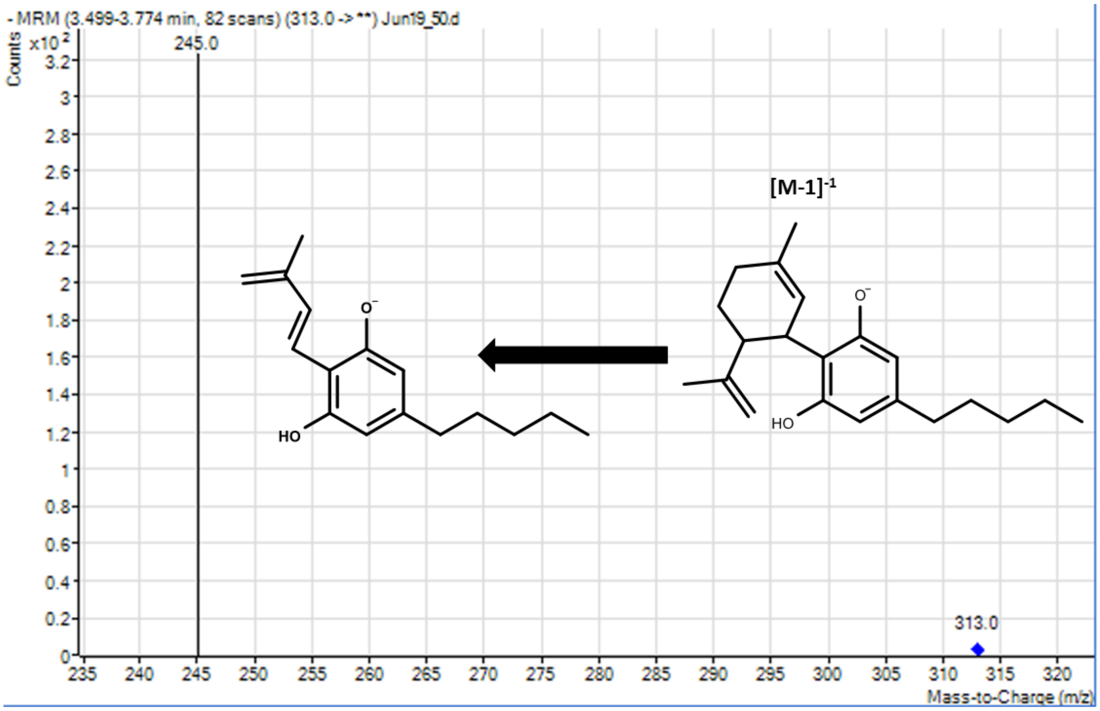

Supplementary Figure S1. The potential fragmentation of CBD in negative electrospray ionization and the characteristic MRM mass spectrum of CBD (313 *m*/*z* → 245 *m*/*z*, collision energy: 20 eV)

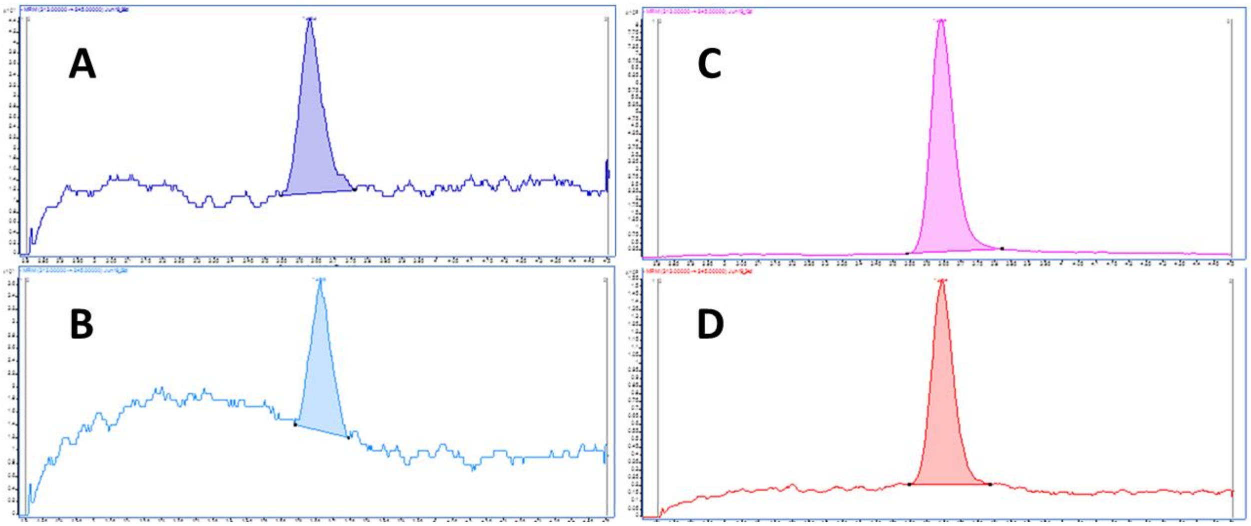

Supplementary Figure S2. The chromatograms obtained at the quantification limits (A: plasma with 1 ng/mL CBD and B: homogenized brain sample with 1 ng/ml CBD) and the representative chromatograms of the real samples (C: plasma with 47.3 ng/ml CBD and D: homogenized brain sample with 6.0 ng/ml CBD).

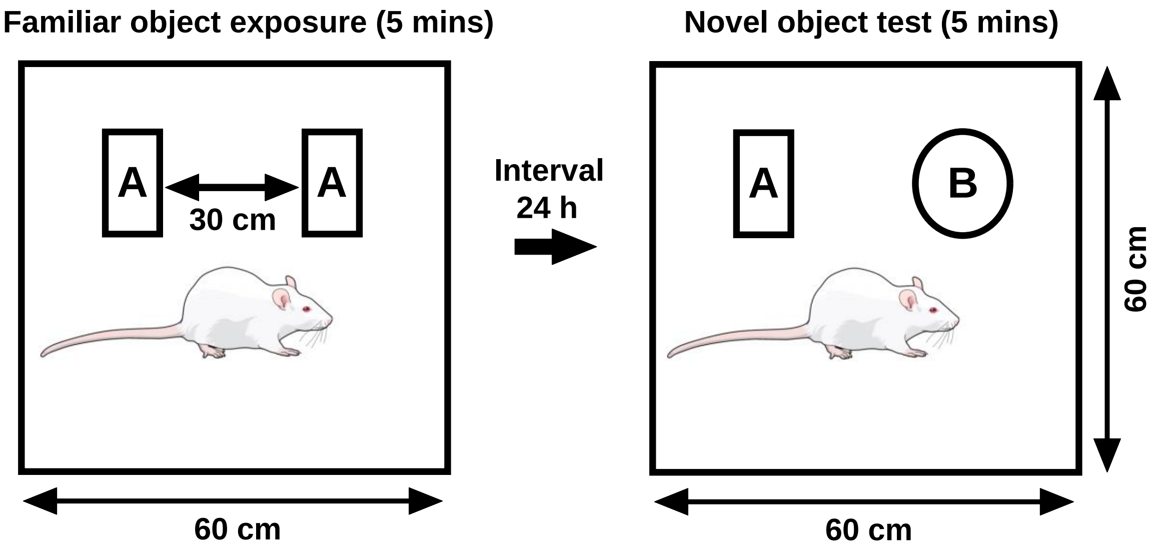

Supplementary Figure S3. Schematic illustration of the novel object recognition task. Rats were habituated to the empty arena and then familiarized with two identical objects (left) for 5 minutes. After a specific interval (24 h), a novel object was presented to each rat and their exploratory behavior was analyzed.

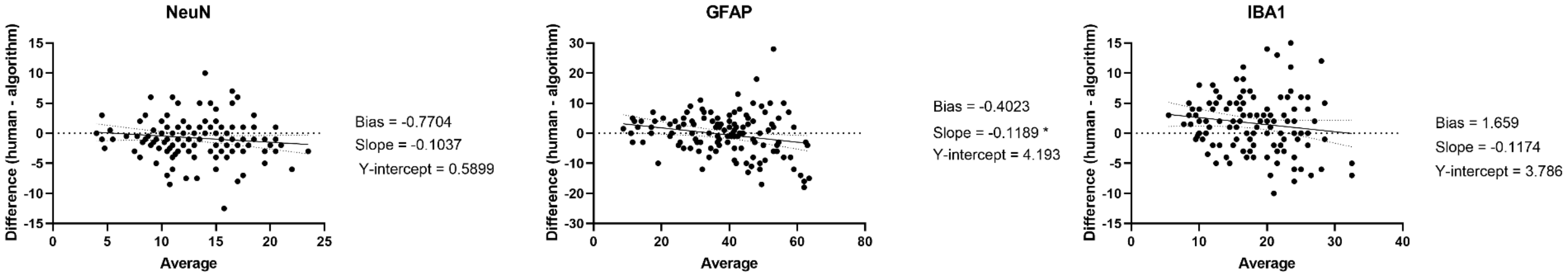

Supplementary Figure S4. Bland–Altman plots representing the agreement between the cell counter algorithm and human manual counting of NeuN-, GFAP-, and IBA1-positive cells in the rat hippocampus. The solid and dashed lines represent the bias and its trend that affect measurement process, and the dotted lines represent the theoretical perfect agreement between the two different counting methods.

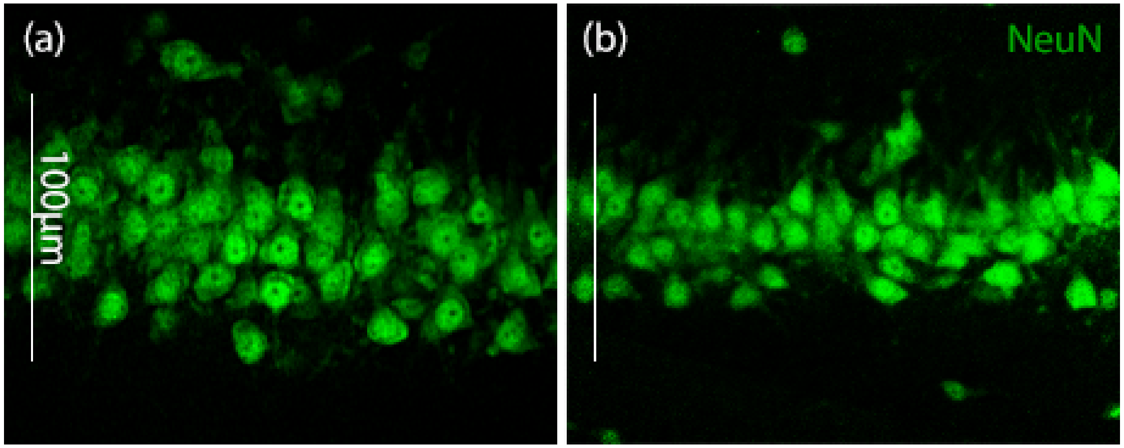

Supplementary Figure S5. Representative image of morphological changes in the CA3 hippocampal pyramidal layer of (a) PTZ-kindled controls and (b) CBD-treated rats.
